# Odd–Even Alkyl Chain Effects on the Structure and Charge Carrier Transport of Two-Dimensional Sn-Based Perovskite Semiconductors

**DOI:** 10.1021/jacs.4c03936

**Published:** 2024-07-02

**Authors:** Shuanglong Wang, Mukunda Mandal, Heng Zhang, Dag W. Breiby, Okan Yildiz, Zhitian Ling, George Floudas, Mischa Bonn, Denis Andrienko, Hai I. Wang, Paul W. M. Blom, Wojciech Pisula, Tomasz Marszalek

**Affiliations:** † Max Planck Institute for Polymer Research, Ackermannweg 10, Mainz 55128, Germany; ‡ Department of Physics, Norwegian University of Science and Technology (NTNU), Høgskoleringen 5, 7491 Trondheim, Norway; § Department of Physics, University of Ioannina, P.O. Box 1186, Ioannina 451 10, Greece; ∥ Nanophotonics, Debye Institute for Nanomaterials Science, Utrecht University, Princetonplein 1, CC Utrecht 3584, The Netherlands; ⊥ Department of Molecular Physics, Faculty of Chemistry, Lodz University of Technology, Zeromskiego 116, Lodz 90-924, Poland

## Abstract

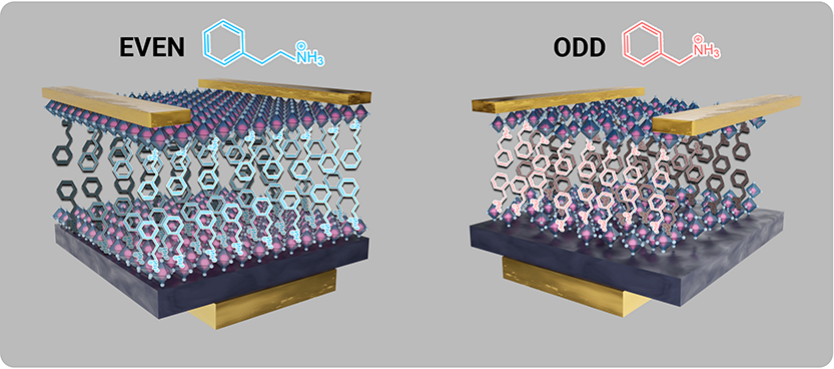

Oscillations in the chemical or physical properties of materials, composed of an odd or even number of connected repeating methylene units, are a well-known phenomenon in organic chemistry and materials science. So far, such behavior has not been reported for the important class of materials, perovskite semiconductors. This work reports a distinct odd–even oscillation of the molecular structure and charge carrier transport properties of phenylalkylammonium two-dimensional (2D) Sn-based perovskites in which the alkyl chains in the phenylalkylammonium cations contain varying odd and even carbon numbers. Density functional theory calculations and grazing-incidence wide-angle X-ray scattering characterization reveal that perovskites with organic ligands containing an alkyl chain with an odd number of carbon atoms display a disordered crystal lattice and tilted inorganic octahedra accompanied by reduced mobilities. In contrast, perovskites with cations of an even number of carbon atoms in the alkyl chain form more ordered crystal structures, resulting in improved charge carrier mobilities. Our findings disclose the importance of minor changes in the molecular conformation of organic cations have an effect on morphology, photophysical properties, and charge carrier transport of 2D layered perovskites, showcasing alkyl chain engineering of organic cations to control key properties, of layered perovskite semiconductors.

## Introduction

Odd–even effects refer to an alternating variation of structure and properties in biological and synthetic systems depending on the odd or even number of structural molecular units. The most prominent examples are alkane derivatives that pack more efficiently when composed of an even number of carbons than those with an odd carbon number due to stronger van der Waals interactions.^1,2^ This phenomenon arises from different orientations of the terminal methyl group, depending on the parity of the number of carbon atoms. In alkanes, this effect typically induces an alternating melting point with higher temperatures observed for even-numbered molecules.^3^ The odd–even effect extends beyond alkane derivatives and has been observed for various alkyl-substituted molecules, such as liquid crystals and recently reported organic semiconductors. These materials exhibit oscillating thermal properties, crystal structure, or photophysical behavior attributed to intermolecular contacts at the termini of odd- or even-numbered side chains.^4−7^ For example, the molecular packing, phase transitions, and charge carrier transport of 2-monoalkylated-benzothieno[3,2-*b*][1]benzothiophenes alternate with the odd–even parity of the alkyl chain length due to variations in the chain–chain interactions.^8^

While odd–even alkyl chain effects have been reported for a wide variety of molecular systems, this phenomenon has not yet been observed for the important class of perovskite semiconductors. Two-dimensional (2D) layered perovskites with self-assembled alternating organic–inorganic layered structures are based on bulky organic spacer cations.^9,10^ With the rapid progress in the field of 2D perovskite light-emitting diodes and photovoltaics, it has been impressively demonstrated that organic ligands with tailored chemical structures provide an effective strategy to fine-tune their electronic properties and thus optimize the optoelectronic devices.^11,12^ One important strategy in the structure modification of the 2D layered perovskites is the incorporation of alkyl-substituted cations. In recent years, several groups have reported the influence of the cation alkyl chain length on device performance. For instance, a higher external quantum efficiency of light-emitting diodes was observed for quasi-2D perovskites containing phenylalkylammonium-based cations with longer alkyl chains that established strong hydrogen bonding with the formamidinium cation.^13^ In another study, a series of linear aliphatic alkylammonium cation spacers with different chain lengths were investigated in quasi-2D perovskite solar cells, and the device efficiency was shown to increase with increasing the length of cations.^14^

Despite this progress, it is worth noting that far fewer studies have focused on the effect of alkyl chain length on charge carrier transport properties of perovskite semiconductors. Li et al. combined both sum frequency generation vibrational spectroscopy and optical-pump terahertz-probe spectroscopy to investigate the charge dynamics in a 2D layered Pb-based perovskite with linear alkyl ligands. It was reported that the charge mobility of 2D perovskites decreased with increasing the length of spacer cations.^15^ While this study provided the first qualitative report on the role of alkyl length on tuning electrical effects, how precisely the chain length of the ligand affects the underlying structural and electronic properties of perovskites remains elusive. Field-effect transistors (FETs) represent an ideal platform to investigate the long-range charge carrier transport properties in perovskite semiconductors, including the influence of interfaces and morphology, which are critical for device integration.^16−21^ Moreover, while the studies mentioned above have investigated the effect of chain length on perovskite properties, none of them have systematically investigated odd–even effects in the alkyl chain.

In this work, we focus on the role of the alkyl chain length of organic spacer cations in the crystal structure and charge carrier transport of 2D layered perovskites. We introduce a series of phenylalkylammonium-based organic spacer cations with different alkyl side chains, namely, phenylmethylammonium (PMA), phenethylammonium (PEA), phenylpropylammonium (PPA), and phenylbutanammonium (PBA), respectively, in 2D layered Sn-based perovskite thin films. Remarkably, the photophysical behavior, structure, and charge transport properties of the corresponding 2D layered perovskites strictly depend on the odd–even parity of the alkyl-chain length, as observed previously for various molecular systems. The perovskites with PMA and PPA cations of odd carbon numbers exhibit an extremely low charge mobility in FETs. On the contrary, devices based on (PEA)_2_SnI_4_ and (PBA)_2_SnI_4_ with even carbon numbers of the ligands result in pronounced gate modulation and field-effect mobilities of 0.33 and 0.17 cm^2^V^–1^s^–1^ at room temperature. This odd–even oscillation in the conductivity is further confirmed by optical pump-terahertz (THz) probe (OPTP) spectroscopy measurements. To understand the origin of the effect in these layered perovskites, the distortion of the crystal lattice is analyzed by combining density functional theory (DFT) calculations as well as experimental and simulated grazing-incidence wide-angle X-ray scattering (GIWAXS) characterizations. We have found that odd carbon numbered PMA- and PPA-based perovskites form tilted octahedral units with larger effective mass values and a disordered structure, resulting in inferior carrier transport. The above results indicate that the previously largely neglected alkyl chain length of spacer cations plays a critical role in controlling charge carrier transport in 2D layered perovskite FETs. Our findings provide a molecular-level understanding of the role of organic cations in optimizing 2D perovskite FETs and, to the best of our knowledge, for the first time, disclose the odd–even alkyl chain effect in perovskites.

## Results and Discussion

**Figure 1:**
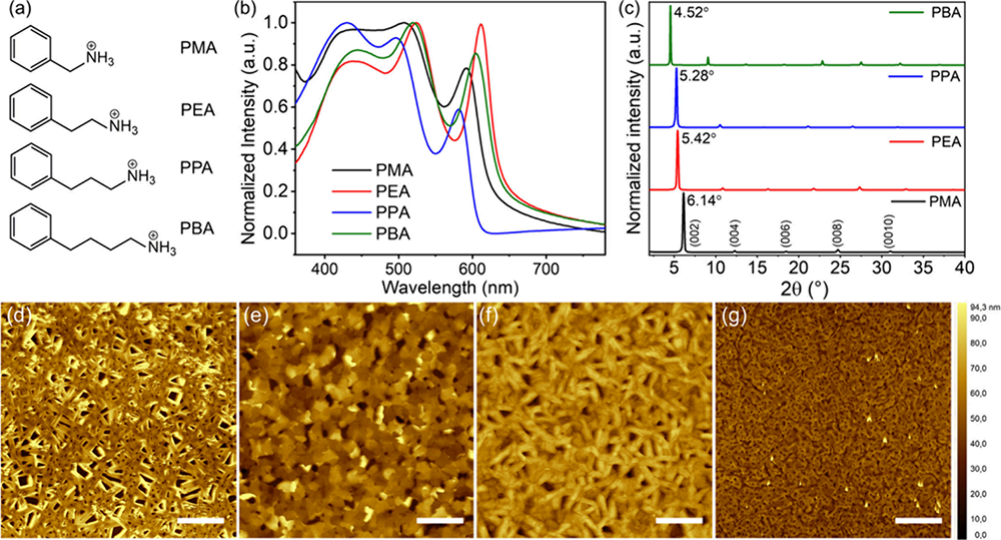
(a) Chemical structures of the four cations PMA, PEA, PPA, and PBA. (b) Ultraviolet–visible absorption spectra. (c) XRD patterns and AFM height images of the corresponding: (d) PMA, (e) PEA, (f) PPA, and (g) PBA perovskite thin films (scale bar: 10 μm).

The chemical structures of the four spacer cations, PMA, PEA, PPA, and PBA are shown in Figure 1a. The average thickness (*n* = 1) of the inorganic layers of the four investigated perovskites was strictly controlled by the molar ratio of the precursors, which was 2:1 between organic cation and SnI_2_ and the layer number was confirmed by the below XRD results. The optical absorption spectra of perovskite thin films deposited from the different cations bear similar characteristics with three main absorption peaks for a layered perovskite structure.^22^ The third absorption peaks are at 593, 612, 581, and 605 nm for PMA, PEA, PPA, and PBA incorporated perovskites, respectively, are attributed to the intrinsic exciton absorption of the layered perovskite lattice (Figure 1b). Interestingly, the excitonic peaks for the perovskite films with even carbon numbers are generally red-shifted compared to their odd counterparts with one less carbon atom so that the optical band gap of the 2D perovskites reveals a remarkable odd–even oscillation depending on the ligand length. Previous reports indicated that distorted crystal geometries increase the band gap of perovskite semiconductors.^23,24^ Structural parameters such as the octahedral tilt, Sn–I–Sn bond angles, and penetration depth of the spacers were reported to affect the energetic landscape. The crystal structures with the critical structural parameters of these perovskites that widen the band gap will be discussed later in more detail.

Photoluminescence (PL) measurements for the four Sn-based perovskite films were also conducted, and the corresponding spectra in Figure S1 exhibit emission peaks at 630, 638, 605, and 634 nm for PMA, PEA, PPA, and PBA, respectively. A larger broadening of the photoluminescence peak for the PPA-based perovskite is noticed. This could be ascribed to the presence of defects in the distorted inorganic sheets of face-sharing and corner-sharing SnI_6_-octahedra, as also presented later.

Room-temperature X-ray diffraction (XRD) confirmed the out-of-plane molecular organization of the four perovskites. In Figure 1c, all the perovskite films reveal typical (00*l*) (l = 2, 4, 6, 8, 10, 12) diffraction peaks that are indicative of the formation of an ideal *n* = 1 layered structure, in which the organic layer and the inorganic framework alternately stack.^25^ The full-width at half-maximum (fwhm) values of the sharp (002) diffraction peak are on an identical level for the investigated perovskite films as shown in Figure S2, indicating similar crystallinity. The (002) peak located at 6.14°, 5.42°, 5.28°, and 4.52° are related to the interlayer distances between the inorganic layers as 14.4, 16.3, 16.7, and 19.6 Å for PMA-, PEA-, PPA-, and PBA-based perovskite films, respectively. Notably, the interlayer distances of the perovskites show a nonlinear increase with the incorporation of PPA and PBA cations. This indicates that the longer alkyl chain lengths lead to a higher molecular degree of freedom and, thus, to their tilted orientation toward the inorganic [SnI_6_] fragment compared to the shorter alkyl chains.^26^

The film morphology of perovskites, more specifically its uniformity, coverage, and roughness, is another crucial parameter that strongly influences charge transport. Atomic force microscopy (AFM) was employed to evaluate the film morphology, as shown in Figure 1d–g. Interestingly, both PMA- and PPA-based films (cations with an odd number of carbon atoms in the alkyl chain) exhibit a dense network of nanorod-like structures with a diameter of around 1 μm, indicating that the perovskite crystals tend to grow directionally. However, at the same time, pinholes and cracks over the entire film surface are observed. A relatively high root-mean-square (RMS) roughness of 21.3 and 18.6 nm for the PMA- (d) and PPA-based films (f), respectively, is determined. These inferior morphologies of the PMA- and PPA-based films might be detrimental to the device performance due to charge carrier trapping at the grain boundaries. In contrast, a smooth film morphology with full coverage of grains is obtained for the cations with even numbers (PEA – Figure 1e and PBA – Figure 1g), and the surface roughness is reduced to 8.9 and 6.4 nm, respectively. The line profiles derived from AFM images in Figure S3 show the strongest variation in height for the (PMA)_2_SnI_4_ film compared to other samples, indicative of a large surface inhomogeneity. We hypothesize that the morphological differences are related to variations in crystallization behavior induced by the odd- and even-numbered organic spacers.^27^

**Figure 2:**
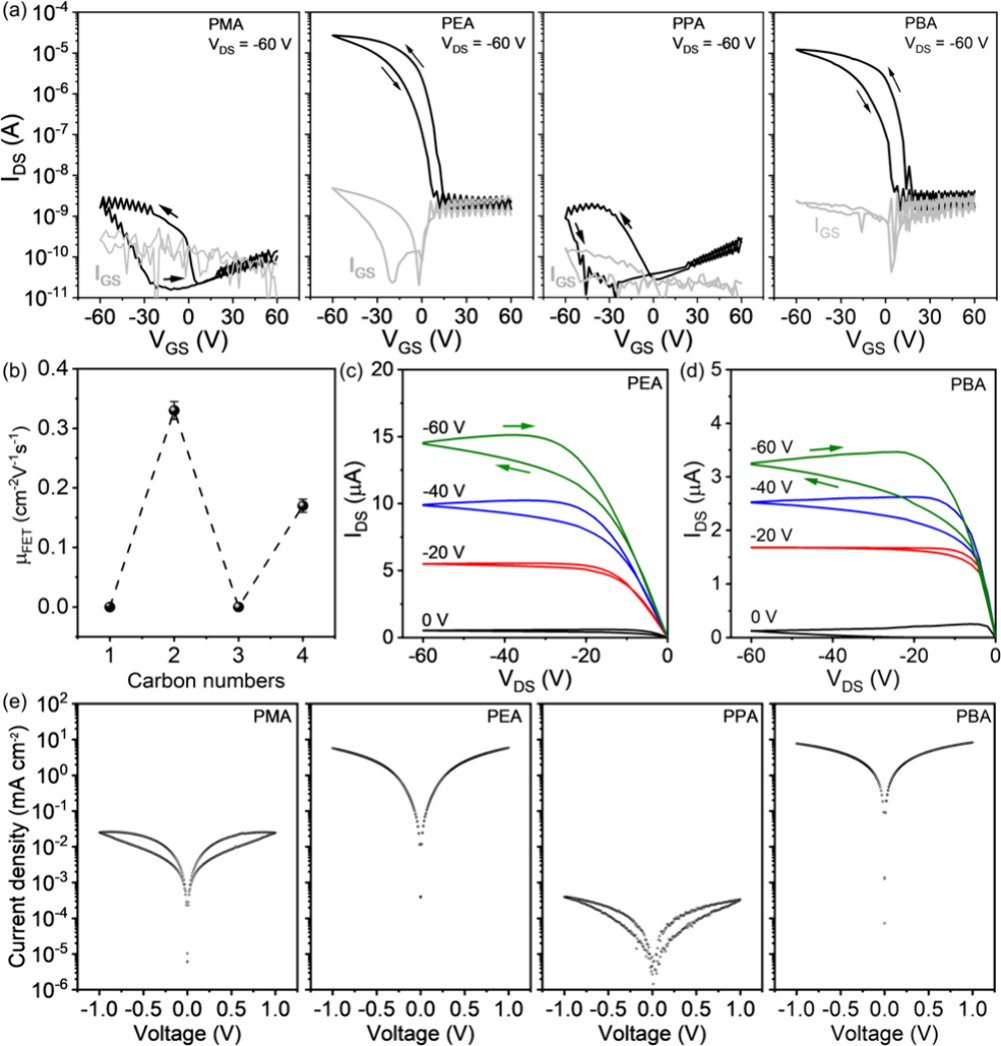
(a) Transfer curves of perovskite FETs based on the cations PMA, PEA, PPA, and PBA at 295 K, respectively. (b) Corresponding μ_FET_ values based on perovskite FETs fabricated from different cations. Error bars indicate the standard deviation from eight devices. Output curves of (c) (PEA)_2_SnI_4_ and (d) (PBA)_2_SnI_4_ FETs at 295 K. (e) Current–voltage diode characteristics of the four perovskite films.

To investigate the in-plane charge carrier transport of the four perovskites, FETs with a bottom-gate and top-contact device configuration with channel length (L) and width (W) of 80 and 1000 μm, respectively, were fabricated (see more details in Methods). Transfer curves were recorded at drain–source voltages (V_DS_) of −60 V with gate–source voltage (V_GS_) scans from +60 V to −60 V. The 2D tin halide perovskite FET devices of the four perovskites show a *p-*type performance, as is evident from the device operation in Figure 2a, with a clear odd–even effect on the electrical parameters. At 295 K, the devices based on PMA and PPA (odd numbers of carbon atoms in the alkyl chain) reveal poor field-effect behavior. On the contrary, FETs based on PEA and PBA (even carbon numbers) exhibit a pronounced performance. Specifically, the (PEA)_2_SnI_4_ FET shows a threshold voltage (V_TH_) of 10 V, on/off current ratio (I_ON/OFF_) ratio of ∼1 × 10^4^, and field-effect mobility μ_FET_ of 0.33 cm^2^V^–1^s^–1^, demonstrating device parameters comparable to previously reported values (Figure 2b).^28,29^ The device based on a (PPA)_2_SnI_4_ channel layer also exhibits a notable electrical performance with a μ_FET_ of 0.17 cm^2^V^–1^s^–1^, V_TH_ of 15 V, and I_ON/OFF_ of 9 × 10^3^. Furthermore, the clear linearity at low V_DS_ and current saturation at high V_DS_ in the output characteristics in Figure 2c and d demonstrate a negligible charge injection barrier between the perovskite channel and source-drain electrodes for both devices.^30,31^ Additionally, the bias stress stability for perovskite FETs based on (PEA)_2_SnI_4_ and (PBA)_2_SnI_4_ with even-numbered organic cations was investigated. Figure S4a shows the change in the source-drain current I_DS_ under a constant bias of V_GS_ = V_DS_ = −60 V for 500 s. A similar sharp decline in the normalized source-drain current (I_DS_(t)/I_DS_(0)) is observed for both FETs. For example, the decay time to reach 50% of the initial channel current is around 58 and 53 s, for (PEA)_2_SnI_4_ and (PBA)_2_SnI_4_ FETs, respectively. The V_TH_ exhibits a shift around 8 V for both perovskites (Figure S4b).

The odd–even effect on the charge transport properties in the out-of-plane direction is also evident in the current density–voltage characteristics of the diode devices in Figure 2e. Similar to the results of FETs, for the devices based on PEA and PBA spacer cations with even carbon numbered alkyl side chains high current density and hysteresis-free current–voltage curves are observed. Devices with odd-numbered PMA and PPA lead to large dual-sweeping hysteresis and much lower current density, indicative of reduced out-of-plane charge transport.

**Figure 3:**
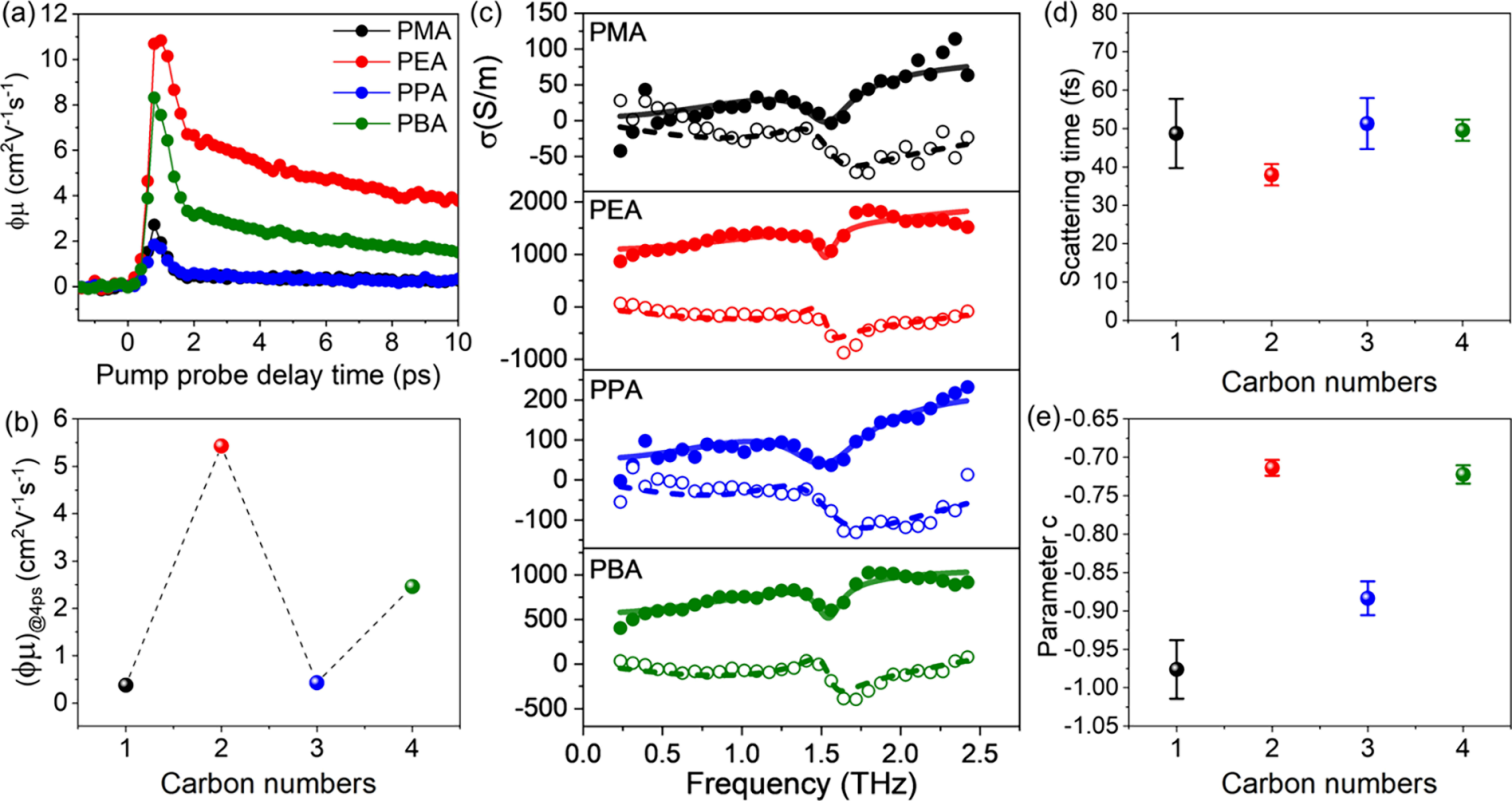
(a) Photoconductivity dynamics under 3.10 eV excitations with an incident pump fluence of 56 μJ cm^–2^. (b) Comparison of the effective mobility at a pump–probe delay time of 4 ps in panel (a). (c) Photoconductivity spectra recorded at a pump–probe delay time of 4 ps. (d, e) Comparison of scattering time τ and parameter *c* for different cations extracted from fits in panel (c).

To confirm that the odd–even alkyl effect on the device performance originates from the intrinsic property of the perovskite films, ultrafast terahertz spectroscopy with subpicosecond time resolution was performed in a contact-free fashion to measure the microscopic photoconductivity (see Method for more details).^32,33^ Different from the FET measurement, THz pulses with ∼ps duration probe and report the charge transport properties within domains of 10s of nm in perovskites. Figure3a shows the photoconductivity dynamics normalized to the absorbed photon densities, which is proportional to the product of charge carrier generation quantum yield ϕ and charge mobility μ, that is, an effective charge mobility ϕμ, for all perovskite films.^34,35^ In spite of similar dynamics for all four perovskites, perovskite films with even-numbered (i.e., PEA and PBA) feature 1 order of magnitude higher ϕμ values in comparison to the odd-numbered samples as illustrated in Figure 3b. This confirms the microscopic origin of the odd–even modulation of the charge carrier transport, as observed in the FET measurements.

To further corroborate the odd–even effect and gain more insight into the microscopic parameters relevant for charge transport, frequency-resolved photoconductivity spectra were recorded at a pump–probe delay time of 4 ps and are shown in Figure 3c for all samples. These spectra are adequately described by combined Drude-Smith model (Δσ_*DS*_, accounting for the free carrier response) and Lorentz model (Δσ_ph_, accounting for the phonon mode at ∼1.6 THz clearly apparent from the steady-state terahertz response)^36^:σ(ω) = Δσ_DS_ + Δσ_ph_ (see Method). From the fitting, the scattering time τ and parameter *c* are obtained as shown in Figure 3d-e. The parameter *c*, ranging between 0 and −1, describes the backscattering probability or the extent of confinement of charge carriers due to, e.g., the presence of structural disorder or grain boundaries. While the charge scattering time shows little variation within experimental uncertainty (Figure 3d), the oscillation of parameter *c* in Figure 3e underlines the photoconductivity oscillation shown in Figure 3a,b. In particular, the parameter *c* for cations with odd carbon numbers is much closer to −1 indicating a stronger confinement of charge transport in these samples. We assign this to increased structural disorder, which will be further corroborated in the following sections.

In exploring the structure–property relationship of these layered 2D hybrid perovskites and elucidating the origin of the odd–even alkyl chain effect on the charge carrier transport, a thorough understanding of their crystal structures is crucial. We used computational modeling to generate candidate structures of these four perovskites. Subsequently, we simulated GIWAXS patterns for the generated conformers and compared them to experimentally observed GIWAXS results to identify the most promising structures. In the main text, we highlight the theoretical structures that exhibit the closest resemblance to the experimental data for each system, while a comprehensive overview of all explored structures is available in the Supporting Information (Supplementary Figures S5–S7).

**Figure 4:**
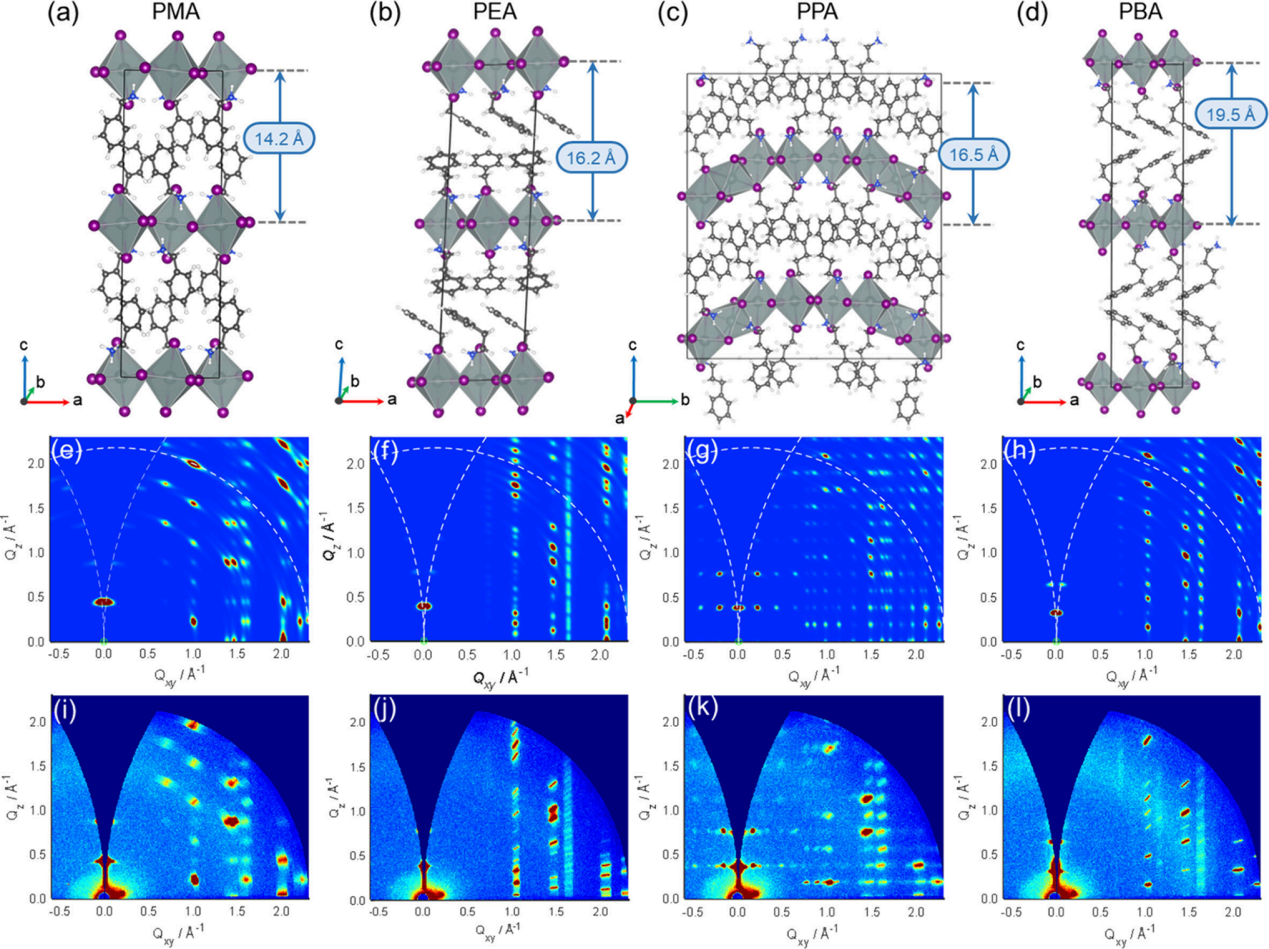
2D perovskite structures explored in the study. (a,d) Models for the four 2D perovskites studied are presented. The chain length of the organic spacer was varied by altering the number of aliphatic carbon atoms between the phenyl ring and the NH_3_^+^ headgroup. The distance between adjacent inorganic layers is also shown for each structure. (e,h) Simulation and (i,l) experimental GIWAXS patterns of the 2D perovskite structures are presented.

The crystal structure of (PMA)_2_SnI_4_ has been previously characterized by Mao et al.^37^ We adopted this structure as our starting point for further optimization using DFT (Figure 4a). The simulated GIWAXS pattern using this candidate structure shows excellent agreement with the experimentally obtained GIWAXS pattern, as depicted in Figures 4e and 4i, respectively. In the case of (PEA)_2_SnI_4_, two different crystal structures have been reported in the literature.^23,38−40^ We explored both structures and simulated the corresponding GIWAXS patterns. In our simulation, the GIWAXS pattern obtained (Figure 4f) using the structure reported by Gao et al. as the initial guess (Figure 4b) demonstrates better agreement with the experimental GIWAXS result (Figure 4j).^38,39^ Therefore, we present this conformer in the main text, while other structures are provided in the Supporting Information Figures S5–S7.

For the systems involving longer organic spacers, PPA and PBA, we drew inspiration from the study of Kamminga et al. on Pb-based layered hybrid perovskites with analogous organic cations.^41^ By substituting Pb with Sn and optimizing both structures, we obtained the PPA structure featuring both corner- and face-sharing [SnI_6_]-octahedra (Figure 4c), which shows the best correspondence between the simulated and experimental GIWAXS patterns (Figure 4g,k, respectively). Consequently, we chose this structure for further electronic structure analysis. However, a similar comparison between the simulated and experimental GIWAXS patterns led us to discard the analogous structure for the PBA cation. Instead, for PBA-based perovskite, we propose a structure with regular corner-sharing octahedra (Figure 4d), which exhibits better agreement between the simulated and experimental GIWAXS data (Figure 4h,l). In addition to comparing GIWAXS patterns, we utilized the interinorganic layer distance (*d*_int-layer_) as a parameter for screening various DFT-computed structures. The conformers presented in the main text show good agreement between the computed (Figure 4a–d) and experimentally measured (Figure 1c) *d*_int-layer_ values (in Å; experimentally obtained values are in parentheses): 14.2 (14.4), 16.2 (16.3), 16.5 (16.7), and 19.5 (19.6) for PMA-, PEA-, PPA-, and PBA-based perovskites, respectively. The consistency of the calculated and experimental *d*_int-layer_ values further justifies the selection of these conformers as the most probable structures for the investigated systems.

**Figure 5:**
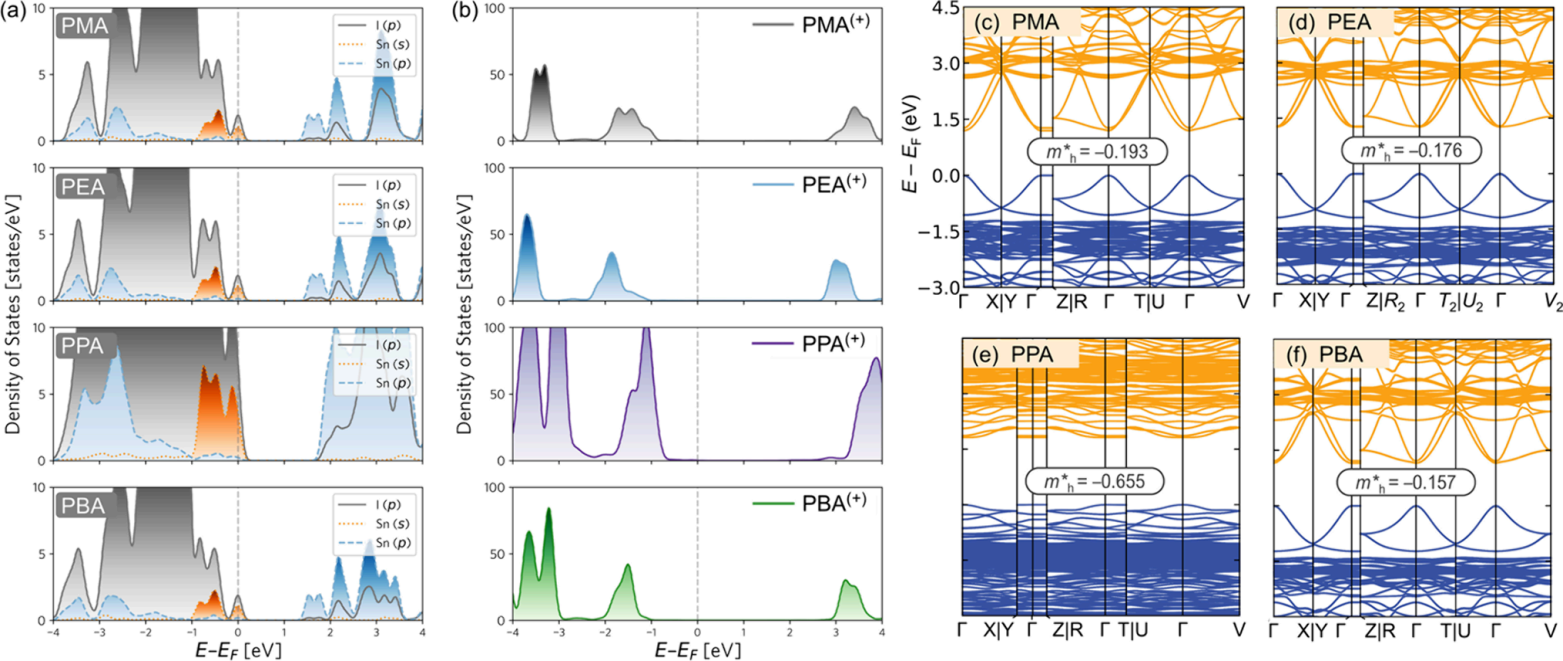
Electronic densities of states (DOS) and band-structures computed using DFT are presented. (a) DOS projected onto I(p), Sn(s), and Sn(p) orbitals near the VBM, depicting the DOS contribution from the inorganic [SnI_6_] layer. (b) Net DOS contribution from the organic spacer, combining orbital contributions from C, H, and N atoms. The corresponding electronic band-structure of (c) PMA-, (d) PEA-, (e) PPA-, and (f) PBA-based perovskites computed using the PBE density functional. The VBM is set to zero in all panels. Effective masses of holes () are reported in the Γ⃗Y direction for all cases, except for PPA, where the value is reported in the Γ⃗X direction. The reported values are in the units of free electron rest mass (*m*_0_= 9.11 × 10^–31^ kg).

DFT calculations were carried out to investigate the influence of the alkyl chain length on the electronic properties of these “well/barrier” composite 2D-perovskites. Figure 5a,b depicts the densities of states (DOS) of the four materials studied. The DOS contribution exclusively from the inorganic constituents of the perovskite material, comprising Sn and I atoms, is shown in Figure 5a. Our analysis reveals that mainly the I *5p* and Sn *5s* orbitals contribute to the valence band maximum (VBM), while the Sn *5p* and I *5p* orbitals make substantial contributions to the conduction band minimum (CBM).

To assess the contribution of the organic spacer alone, we summed over the partial DOS contributions due to C, H, and N atoms and plotted them in Figure 5b. Our findings show that the organic layer does not have a “direct” impact on the electronic properties of the materials, as its contribution is located away from both the VBM and the CBM. Nevertheless, it is crucial to emphasize that the organization and orientation of the organic spacers significantly affect the structural order within the inorganic sheets, eventually leading to the octahedral tilting of the inorganic [SnI_6_] units. This, in turn, has a direct influence on the electronic properties of the material, which we elaborate on in subsequent sections. The electronic band-structures of the four perovskites between high symmetry points of the first Brillouin zone are presented in Figure 5c-f, which reveal that the VBM and CBM occur at the Γ-point in all cases. The calculated direct band gap values are as follows: 1.19 eV (PMA), 1.26 eV (PEA), 1.82 eV (PPA), and 1.15 eV (PBA). It is important to mention that these computed band gap values are significantly underestimated when compared to experimental measurements (1.97, 1.92, 2.04, and 1.93 eV, respectively). This is expected since the nonempirical Perdew–Burke–Ernzerhof (PBE) generalized gradient approximation functional was employed for the calculations.^42−44^

Effective hole masses for these materials were determined by fitting a third-order polynomial to the band edges. This analysis revealed that perovskites featuring the even-numbered alkyl chains placed between the phenyl and amine head groups of the organic cation, such as PEA and PBA, exhibited lower effective hole masses (*m*_h_^*^) compared to their odd-numbered counterparts, PMA and PPA. The computed  values are as follows: –0.176 (Γ⃗Y) for PEA, – 0.157 (Γ⃗Y) for PBA, – 0.193 (Γ⃗Y) for PMA, and −0.655 (Γ⃗X) for PPA-based perovskites ( values are in the units of free electron rest mass, m_0_). The larger effective hole mass of PPA indicates a lower predicted mobility, possibly due to its structural disorder, as evidenced in Figure 4c, showing both corner- and face-sharing octahedra in the inorganic sheet, suggesting a 1D confinement effect. On the other hand, 2D confinement is predicted for perovskites with PEA and PBA spacers having even-numbered alkyl chains, which makes them good candidates for device applications. Besides the contribution of effective mass, the poor performance of perovskites with the odd number may be attributed to structural disorder arising from octahedral tilting (vide infra). It is generally believed that charge carriers in 2D layered perovskites are confined within the octahedral inorganic layers due to the dielectric effect.^22^ The presence of a tilted crystal structure is expected to hinder charge transport, leading to inferior performance of the electronics devices.

**Figure 6:**
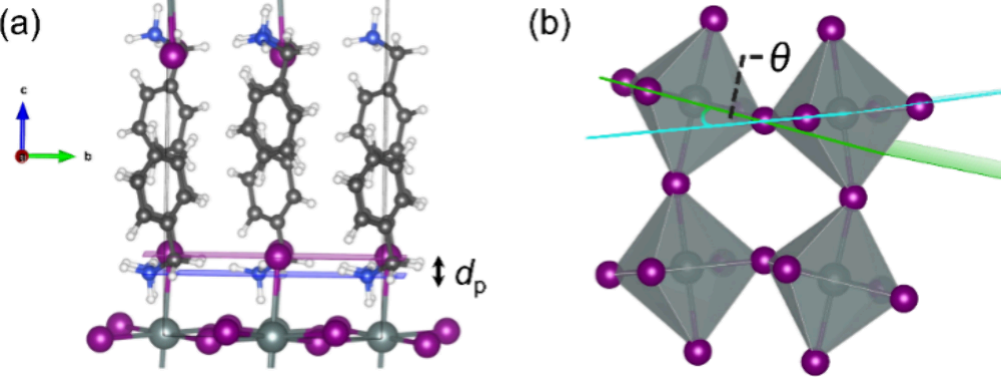
Definition of (a) organic cation penetration depth (d_P_) and (b) distortion angle (θ) of the adjacent octahedral units.

As noted previously, the presence of organic cations significantly affects the structure of the inorganic layer. To better understand the influence of organic cations on the level of disorder within the inorganic sheets, we introduce the parameter organic cation penetration depth (*d*_P_).^45^ This parameter measures the average distance between the plane containing the N atoms of the organic cation and the plane containing the axial I atoms of the inorganic sheet (Figure 6a). A larger *d*_P_ value implies a stronger steric interaction between the organic cation and the inorganic framework, which leads to greater distortion of the octahedral units.^45,46^ This distortion is prominently reflected in the in-plane distortion of the inorganic sheet, indicated by the distortion angle θ (Figure 6b). This angle, measured between the planes containing the planar [SnI_6_] units from two neighboring octahedra, quantifies the extent of structural distortion within each perovskite. A larger distortion angle, θ, signifies a greater deviation of the ∠Sn–I–Sn bond angle from the ideal 180°, indicating more pronounced distortion within the inorganic [SnI_6_]^−^octahedra layers.^45^ As shown in Figure S8, we observed a slightly larger *d*_P_ value for PMA (0.77 Å) than that for PEA (0.58 Å). This difference is expected to induce more distortion within the inorganic sheets, as confirmed by a much larger distortion angle in PMA (θ = 11.22°) compared to that in PEA (θ = 3.11°), as shown in Figure S9. These findings support the notion that the inorganic sheet with the PMA cation experiences greater distortion than its counterpart with PEA, leading to inferior electronic properties such as reduced charge carrier mobility. For the longer-chained cations PBA and PPA, the electronic properties of PBA were found to be superior to those of PPA, which can also be attributed to structural changes induced by the organic cation. Despite the penetration depth values being nearly identical (0.67 Å for PPA and 0.65 Å for PBA, in Figure S8), the distortion angle θ in PPA was considerably greater (7.92°) compared to that in PBA (2.59°). This aligns with our earlier observations that the presence of the PPA cation introduces more substantial distortion to the inorganic layer and leads to the formation of both corner and face-sharing octahedra, resulting in 1D confinement and hence inferior electronic properties.

Additionally, the *ab*-plane of the studied perovskite crystals reveals an intriguing pattern (Figures S10–S12). The orientation of the aryl rings in the organic spacers relative to the inorganic layer appears to correlate with the carbon number in the alkyl chain. In PEA and PBA, with even-numbered alkyl chains, the aryl rings tend to align parallel to the inorganic layer. In contrast, for PMA and PPA with odd-numbered alkyl chains, the benzene rings consistently remain perpendicular to the inorganic layer. While this preliminary observation suggests a possible connection between the aryl π-electron density and its proximity to the inorganic layer in PEA and PBA, potentially influencing dielectric confinement, charge delocalization, and transport, further investigation is crucial to substantiate this hypothesis and elucidate the true nature of this potential correlation. It should be noted that this odd–even variation in the molecular configuration of the phenylalkylammonium-based cations in the perovskite lattice has not been observed for the linear alkyl cations. The odd–even effect has not been experimentally and theoretically observed for the linear alkyl cations in our previous study and by other groups.^31,47,48^ Independent of the carbon number, all linear alkyl cations adopt a linear packing configuration in the 2D perovskite lattice in which the nitrogen atom in the anchoring group is aligned along the same axis with the alkyl tail of the spacer.

Combining the THz results and DFT calculation, we find that the tilted perovskite structures with odd carbon numbers of the phenylalkylammonium-based cations lead to large effective hole mass values  and low local charge mobility. Since the electrode distance in the diode devices with the Au/perovskite/Au architecture is much smaller in comparison to FETs and corresponds simply to the film thickness of 300 nm, the impact of film morphology on the out-of-plane charge transport is lower. Due to the distorted inorganic [SnI_6_]-octahedra layers and the low local charge carrier mobility, the diode performance of the odd-numbered PMA- and PPA-based perovskites is significantly reduced in comparison to the more ordered PEA- and PBA-based semiconductors. The higher diode current of (PMA)_2_SnI_4_ in comparison to PPA-based perovskite is attributed to the less tilted inorganic [SnI_6_]-octahedra layers, as proven by the computational modeling and calculated smaller  for (PMA)_2_SnI_4_. In FETs, the distance between source and drain electrodes is much larger, and the role of film morphology becomes more important. Since (PMA)_2_SnI_4_ shows an inferior film morphology with pinholes and randomly oriented grains of small size, its FET performance and the in-plane charge carrier transport are comparable to PPA-based perovskite. The perovskites with even carbon numbers of the organic spacers show a more regular planar and ordered inorganic [SnI_6_]-octahedra layers and surface morphology, contributing to the improved diode performance as well as local and field-effect charge mobilities which are in agreement with the small .

## Conclusions

In summary, we have introduced a series of alkyl-substituted phenylalkylammonium organic cations in Sn-based layered perovskites. Through systematic variation of the alkyl side chain, a distinct odd–even effect on the crystal structure and charge carrier transport is observed regarding the number of carbon atoms in the side chain. The structural characterization and theoretical calculations reveal that the odd–even number of carbons significantly affects the molecular packing arrangements (and thus the macroscopic morphology), accompanied by a distinct variation in effective mass values. Our findings have disclosed the importance of minor changes in the molecular conformation of organic cations on order, photophysical properties, and charge carrier transport of 2D layered perovskites. These insights provide an understanding of the general role of organic cations on the molecular level and provide guidelines for optimizing the electronic properties of perovskite semiconductors.

## Supporting Information Available

Experimental details, materials and methods including DFT calculations, GIWAXS simulation protocol, PL spectra, XRD patterns, AFM analysis, stability of FETs, and illustration of DFT-calculated crystal structures (PDF) for all compounds (PDF)
