# Supplementary material for: Odd–Even Alkyl Chain Effects on the Structure and Charge Carrier Transport of Two-Dimensional Sn-Based Perovskite Semiconductors
Source: J Am Chem Soc. 2024 Jul 2;146(28):19128–36. doi: 10.1021/jacs.4c03936 (PMC11258789; doi:10.1021/jacs.4c03936)
Supplement: Supplementary file 1 — ja4c03936_si_001.pdf [file ja4c03936_si_001.pdf]

Supporting Information

Odd-even alkyl chain effects on the structure and charge carrier transport of two-dimensional Sn-based perovskite semiconductors

Shuanglong Wang[1], Mukunda Mandal[1], Heng Zhang[1], Dag W. Breiby[2], Okan Yildiz[1], Zhitian Ling[1], George Floudas[1,3], Mischa Bonn[1], Denis Andrienko[1], Hai I. Wang[1,4], Paul W. M. Blom[1], Wojciech Pisula[1,5,*] and Tomasz Marszalek[1,5,*]

[1]Max Planck Institute for Polymer Research, Ackermannweg 10, 55128 Mainz, Germany

[2]Department of Physics, Norwegian University of Science and Technology (NTNU), Høgskoleringen 5, 7491, Trondheim, Norway

[3]Department of Physics, University of Ioannina, P.O. Box 1186, 451 10 Ioannina, Greece

[4]Nanophotonics, Debye Institute for Nanomaterials Science, Utrecht University, Princetonplein 1, 3584 CC Utrecht, The Netherlands

[5]Department of Molecular Physics, Faculty of Chemistry, Lodz University of Technology, Zeromskiego 116, 90-924 Lodz, Poland

[*]To whom correspondence should be addressed.

Email: *pisula@mpip-mainz.mpg.de, marszalek@mpip-mainz.mpg.de*

Table of Content

Experimental section Page 3

PL spectra Page 9

XRD patterns Page 10

AFM analysis Page 11

Bias stress stability of FETs Page 12

Calculated layered crystal structures Page 13

Illustration of organic cation penetration depth Page 16

Calculated inorganic layer distortion angle Page 17

Calculated orientation of organic spacers Page 18

References Page 21

Experimental and Methods Section

Materials

The organic salts phenylmethylammonium iodide (PMAI), phenethylammonium iodide (PEAI), phenylpropylammonium iodide (PPAI) and phenylbutanammonium iodide (PBAI) were all ordered from Xi'An Polymer Light Technology Corp (Xi'An, China). Tin (II) iodide (SnI_2, 99.999%), N,N-dimethylformamide (DMF, anhydrous, 99.8%), dimethyl sulfoxide (DMSO, anhydrous, ≥99.9%) were purchased from Sigma-Aldrich. All the chemicals were used as received without any other refinement unless otherwise specified. Heavily doped Si/SiO_2 (300 nm) Substrates were obtained from Ossila Ltd.

FET fabrication

Bare Si/SiO_2 wafers were cleaned in an ultrasonication bath with acetone and isopropanol for 20 min in a sequential manner. After drying by flowing nitrogen, the substrates were treated with UV/ozone for 20 min and then transferred into a glove box for further use. The perovskite precursor solution with a concentration of 0.15 M was prepared by dissolving the organic cations and SnI_2, with the standard molar ratio of 2:1 in mixed DMF/DMSO (1:1) and kept stirring overnight at room temperature in N_2 glovebox and filtered with a 0.2 μm polytetrafluorethylene filter before use. Perovskite thin films were deposited by spin coating the solution at 4000 rpm for 60 s, followed by thermal annealing at 100 °C for 30 min on a hotplate. Finally, source and drain electrodes with 80-nm gold were thermally evaporated through a shadow mask, constructing 80 × 1000 μm (length × width) transistor channels.

Perovskite film and device characterization

UV-vis Spectroscopy: The optical absorption spectra of perovskite films were measured using an ultraviolet/visible (UV/Vis) spectrophotometer from a PerkinElmer Lamda 25 instrument with an all-reflecting, double-monochromator optical system and holographic gratings used in each monochromator for the UV-vis range. Data was collected from 300 - 800 nm using a dwell time of 0.1 s. During all measurements, the samples were kept in the air and at room temperature.

PL Spectroscopy: Photoluminescence spectra were recorded at room temperature using the Fluorolog-3 with an integrating sphere (F-3018 from Horiba Jobin Yvon) under nitrogen flow to avoid degradation of the perovskite samples.

AFM measurement: The film morphology was characterized by Bruker Dimension Icon FS AFM in tapping-mode at a resonant frequency of 300 kHz and a spring constant of 26 N/m. The root mean square (RMS) roughness values were extracted from an image of 50 μm × 50 μm.

XRD measurements: The room temperature X-ray diffraction patterns of the 2D perovskite thin films were recorded in the 2θ between 3° and 40°, with a step of 0.01° and a speed of 10 degrees per minute, using a Rigaku SmartLab HR-XRD equipment with Cu Kα (λ = 1.5406 Å) radiation. The interlayer distance was calculated by Bragg's law $2dsin\theta = n\lambda$.

GIWAXS characterization: GIWAXS measurements were performed by means of an X-ray tube (Siemens Kristalloflex X-ray source, copper anode X-ray tube operated at 35 kV and 20 mA), Osmic confocal MaxFlux optics, pinhole beam collimation, and a MAR345 image plate detector. The fixed incidence angle was approximately 0.2°. All X-ray scattering measurements were performed under vacuum (∼1 mbar) to reduce air scattering and prevent degradation of the perovskite samples. For direct comparisons with the simulated scattering patterns, the experimental raw data were interpolated to rectilinear (Q_{xy}, Q_z) cylindrical coordinates.

FET device measurement: All devices were characterized using Keithley 4200-SCS operated with pulsed mode in a vacuum of 10^{-6} mbar. Transfer characteristics were operated in the pulsed mode and V_g was applied over a short impulse of 1s. For output measurement, the continuous mode was used and the voltages were applied continuously during the scan. The temperature-dependent transport measurements were performed from 100 K to 295 K in a Janis probe station connected to a cryostat and cooled with liquid N_2. The temperature was monitored using thermocouples. All the electrical

measurements were carried out in a vacuum of 10^{-6} mbar. Mobility values of FETs are extracted with the following equation[1]:

$$\mu = \frac{2L}{WC_i} (\frac{\partial\sqrt{I_{ds}}}{\partial V_g})^2$$

where L, W, and C_i are the channel length and width and the capacitance per unit area of the oxide dielectric, respectively.

J-V measurement*:* For *J-V* electrical characterization, the devices with sandwich structure of Au/Perovskite/Au were fabricated with shadow mask-patterned electrode based on the above-mentioned fabrication procedure accordingly. Thicker perovskite films with the thickness more than 300 nm were employed to minimize leakage current. The measurements were carried out under nitrogen atmosphere in glovebox with a Keithley 2400 source meter.

DFT Calculation Protocol

The computational methodology employed in this study involved first-principles calculations based on density functional theory (DFT) implemented in Vienna Ab-initio Simulation Package (VASP, version 6.3.1).[2] The calculations utilized the generalized gradient approximation (GGA) in the Perdew–Burke–Ernzerhof (PBE) functional form and incorporated Van der Waals interactions via Grimme's D3 method.[3,4,5] The projector augmented wave (PAW) method was utilized to describe valence-core interactions.[6] The calculations assumed the following numbers of valence electrons for each atom type: Sn (*14*), I (*7*), C (*4*), H (*1*), N (*5*). A plane-wave energy cutoff of 520 eV was employed for all calculations. During geometry relaxation, forces of each atom below 0.01 eV/Å were used. The structural relaxation and densities of states (DOS) calculations were both carried out by sampling the Brillouin zone on a 4 × 4 × 1 *k*-mesh (Monkhorst-Pack) in all cases except for the PPA (conformer 1) structure. Because of its larger unit cell, a 4 × 2 × 2 *k*-mesh was used for the PPA case. The VESTA and Sumo packages were utilized for structure visualization and projected DOS, respectively.[7,8] Effective hole masses were calculated from polynomial fitting of the band structures employing the

VASPKIT utility.[9]

GIWAXS simulation

The GIWAXS simulations were carried out using the software SimDiffraction, which is developed to provide accurate calculated scattering patterns for textured polycrystalline thin films, based on long-range ordered (crystalline) molecular structures.[10] Note that compared with for example standard powder diffraction, several factors (including the asymmetric scattering geometry, the long beam footprint on the sample, the pronounced sample anisotropy, and refraction effects) make it challenging to carry out a full structural refinement for these complex molecules based on GIWAXS data. Consequently, an analysis pathway was chosen where density functional theory (DFT, see below) was used to propose several (up to ~10) candidate unit cell structures for each perovskite.[11,12] (For the $(PMA)_2SnI_4$ perovskite, the crystal structure previously published by Mao *et al* was found to give an excellent fit to our data[13]).

Based on the resulting candidate molecular crystal structures specified in CIF files generated from the theoretical modeling, the GIWAXS patterns were predicted, taking into account the pronounced preferred uniaxial orientation of the crystallites. In the present cases, the *a*-*b* plane of the proposed unit cells was found to be highly parallel to the substrate surface. An orientational distribution of a few degrees ($< 4^o$ FWHM) about the surface normal was included in the simulations, understood to model both divergence in the incoming beam, and actual slight crystallite alignment variations. The simulations were convoluted with a fixed radial peak width of 0.03 $Å^{-1}$, accounting for crystal size broadening and the non-monochromatic nature of the beam and other peak broadening effects. GIWAXS is highly sensitive to structural modifications and essentially gives a “fingerprint” containing information about the unit cell dimensions and orientation, the atomic structure, and the degree of preferred orientation, as revealed through the diffraction peak positions, widths, and relative intensities. Referring to Figure 4 in the main text, it is clear that for all four perovskites, there is good

agreement for all these parameters, indicating that the reported structures are correct.

Ultrafast THz time-domain (THz-TDS) spectroscopy

The THz setup is driven by a regenerative amplified, mode-locked Ti:Sappire laser with repetition rate of 1 kHz, central wavelength of 800 nm, pulse duration of 50 fs. The output laser beam is separated into three branches: one for THz generation through optical rectification in a 1 mm-thick ZnTe crystal, one for electrooptic sampling in another 1 mm-thick ZnTe crystal, and one for optical excitation (i.e., pump). In the optical pump-THz probe experiment, a photoexcitation with a photon energy of 3.10 eV generated by a beta barium borate (BBO) crystal was firstly used to generate electrons and holes in the conduction and valence bands, respectively. Subsequently, a single-cycle THz pulse with ~1 ps duration transmitted collinearly through the perovskite thin film on fused silica substrate interacting with the photoexcited charge carriers. This interaction led to the attenuation $\Delta E(t)$ of the incident THz electric field $E(t)$, which is related to the real part of the photoconductivity $\Delta\sigma$ ($\propto -\Delta E/E$).[14]

To describe the photoconductivity spectra and take into account the resonances at ~1.6 THz, we employed the combination of Drude-Smith (DS) mode and Lorentz model: $\sigma(\omega) = \Delta\sigma_{DS} + \Delta\sigma_{ph}$. The DS model is a modified Drude model considering the effect of the charge backscattering, which reads:

$$\sigma(\omega) = \frac{\omega_p^2 \varepsilon_0 \tau}{1 - i\omega\tau}\left(1 + \frac{c}{1 - i\omega\tau}\right)$$

Here ω is the angular frequency, ε_0 is the static dielectric constant, ω_p is the plasma frequency, τ is the DS scattering time. The parameter c (-1<c<0) accounts for the charge backscattering effect due to e.g., the polycrystalline boundaries and lattice distortions.[14,15] When c=-1, the charge carriers are subjected to a complete preferential backscattering resulting in a charge localization; when c=0, the DS model recovers the classical Drude model where the charges experience momentum-randomizing scattering events.

The Lorentz model describes the resonant excitations in the spectra and is given by the formula:

$$\sigma(\omega) = \frac{\omega_p^2 \varepsilon_0 \omega}{i(\omega_0^2 - \omega^2) + \omega\Gamma}$$

in which ω_0 is the resonant frequency, Γ is the damping rate.

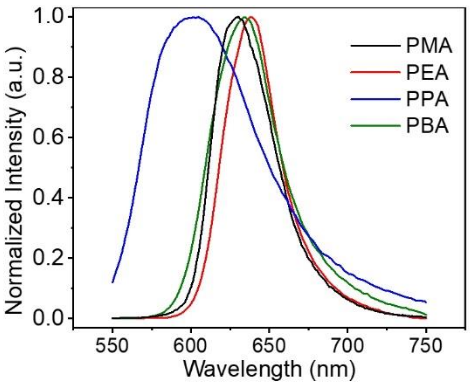

Figure S1. Normalized PL spectra for the 2D Sn-based perovskites based on phenylalkylammonium cations with different alkyl lengths.

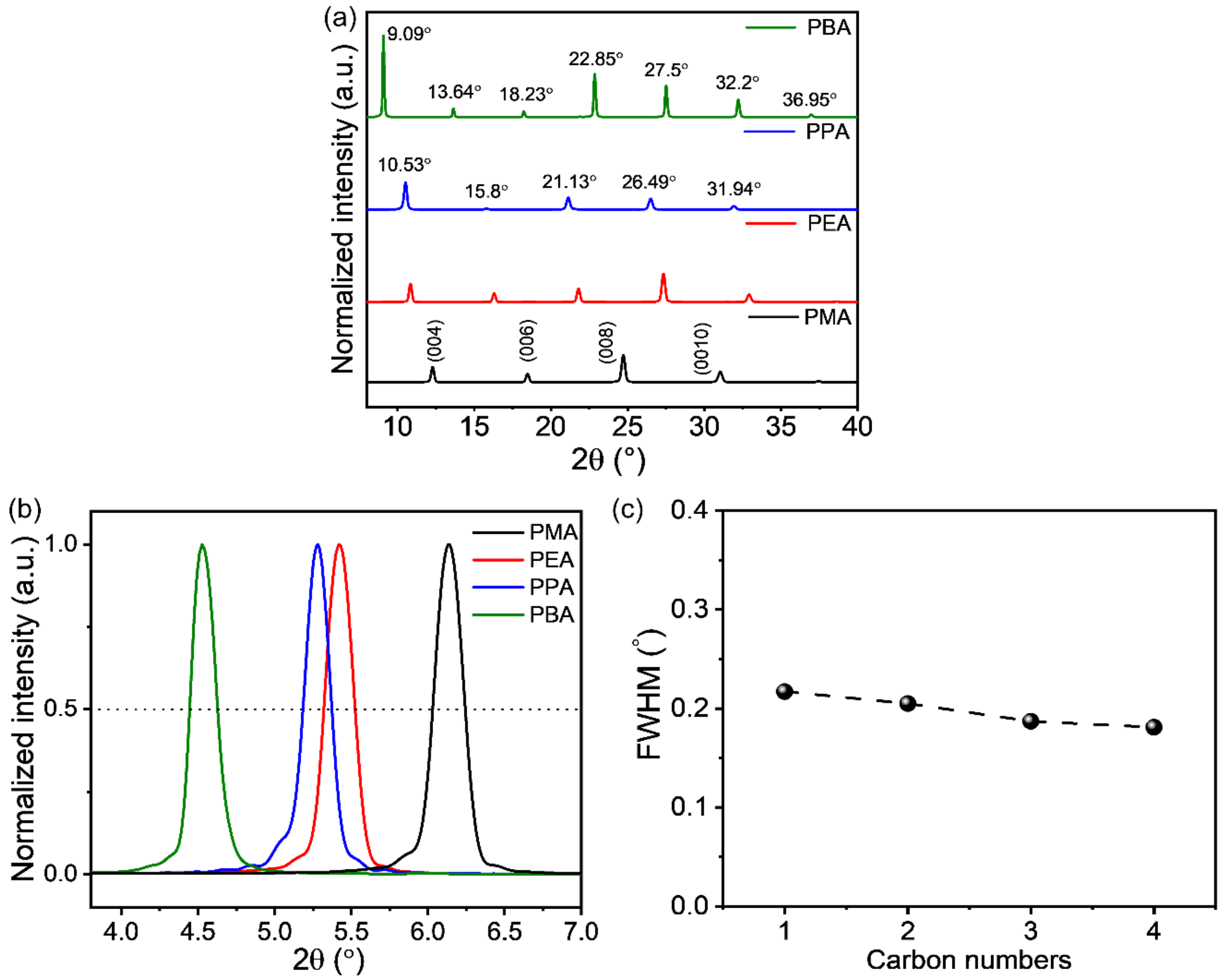

Figure S2. (a) XRD patterns of perovskite films. (b) Full width at half maximum (FWHM) analysis of the (002) peak, and (c) the corresponding FWHM values for the perovskite films as function of carbon number of the spacer cation.

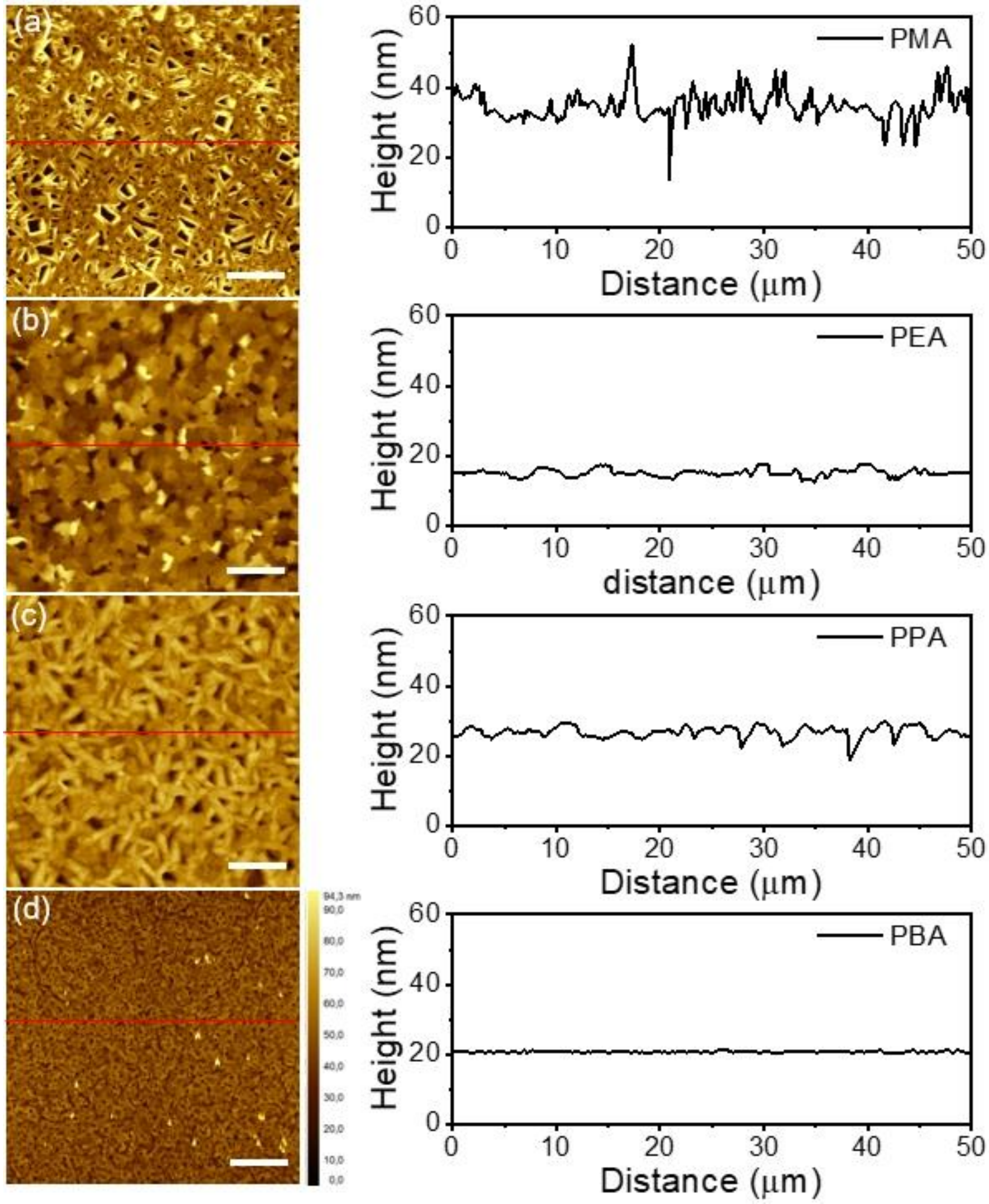

Figure S3. AFM characterization and the corresponding height along scan distance of these four perovskite thin films based on the cation of (a) PMA; (b) PEA; (c) PPA; and (d) PBA.

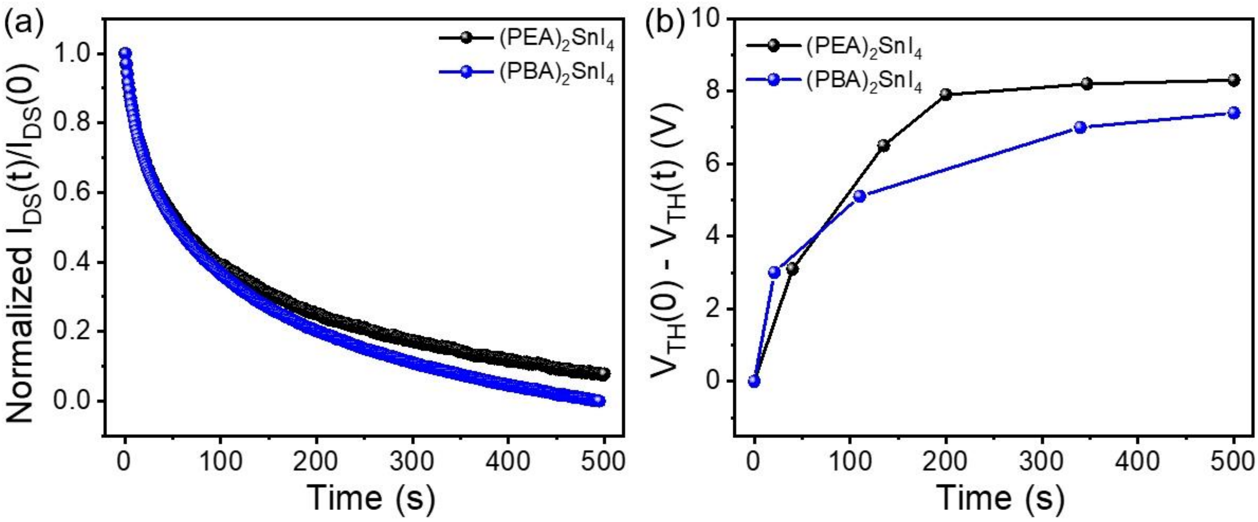

Figure S4. (a) Bias stress stability of $(PEA)_2SnI_4$ and $(PBA)_2SnI_4$ FETs under a constant bias of $V_{GS} = V_{DS} = -60$ V and (b) the corresponding variation of V_{TH}.

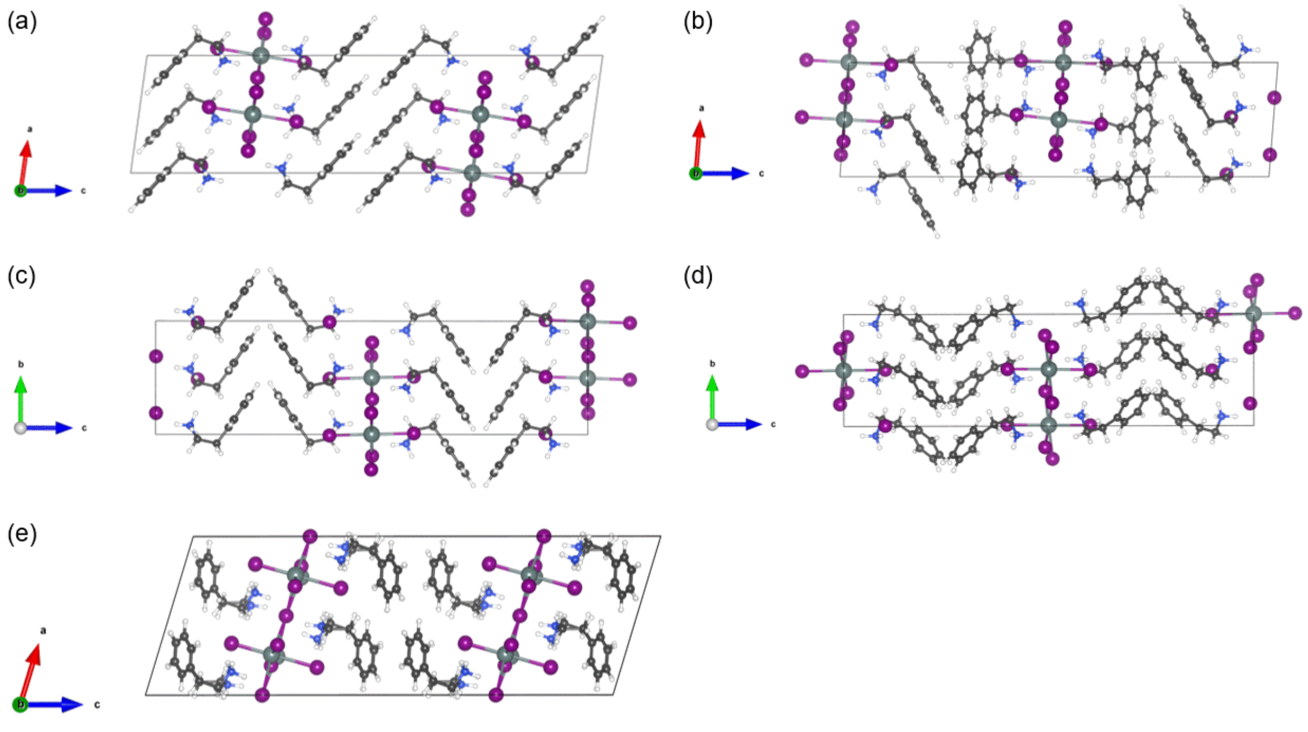

Figure S5. Calculated layered crystal structures based on the organic cation PEA with the interlayer distance of (a) 16.5 Å; (b) 16.2 Å; (c) 16.4 Å; (d) 15.6 Å; and (e) 17.3 Å, respectively.

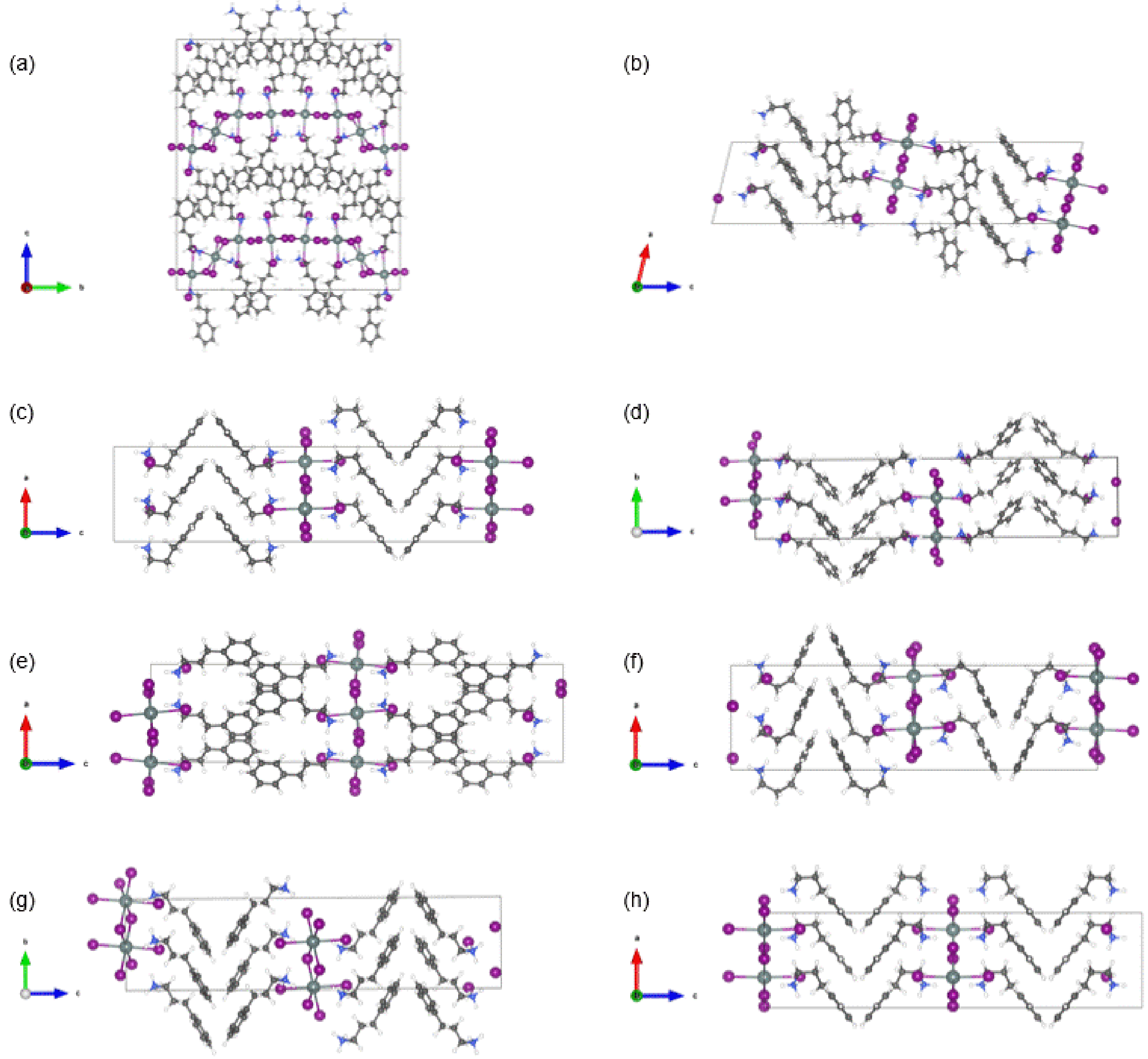

Figure S6. Calculated layered crystal structures based on the organic cation PPA with the interlayer distance of (a) 16.6 Å; (b) 18.1 Å; (c) 17.1 Å; (d) 19.2 Å; (e) 18.8 Å; (f) 16.1 Å; (g) 17.7 Å; and (h) 17.0 Å, respectively.

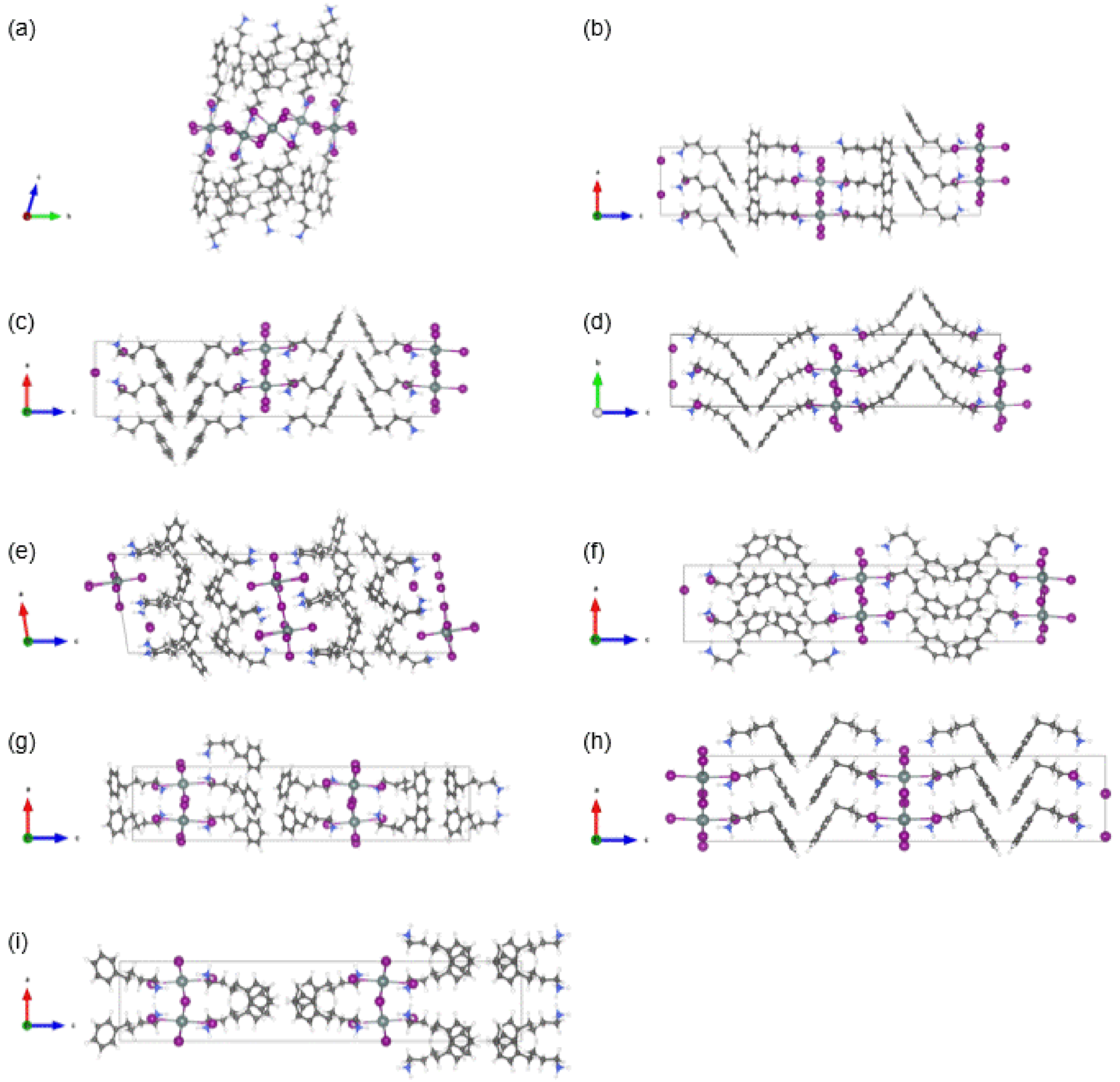

Figure S7. Calculated layered crystal structures based on the organic cation PBA with the interlayer distance of (a) 17.7 Å; (b) 20.4 Å; (c) 19.5 Å; (d) 19.4 Å; (e) 19.6 Å; (f) 19.9 Å; (g) 19.6 Å; (h) 20.2 Å; and (i) 21.3 Å respectively.

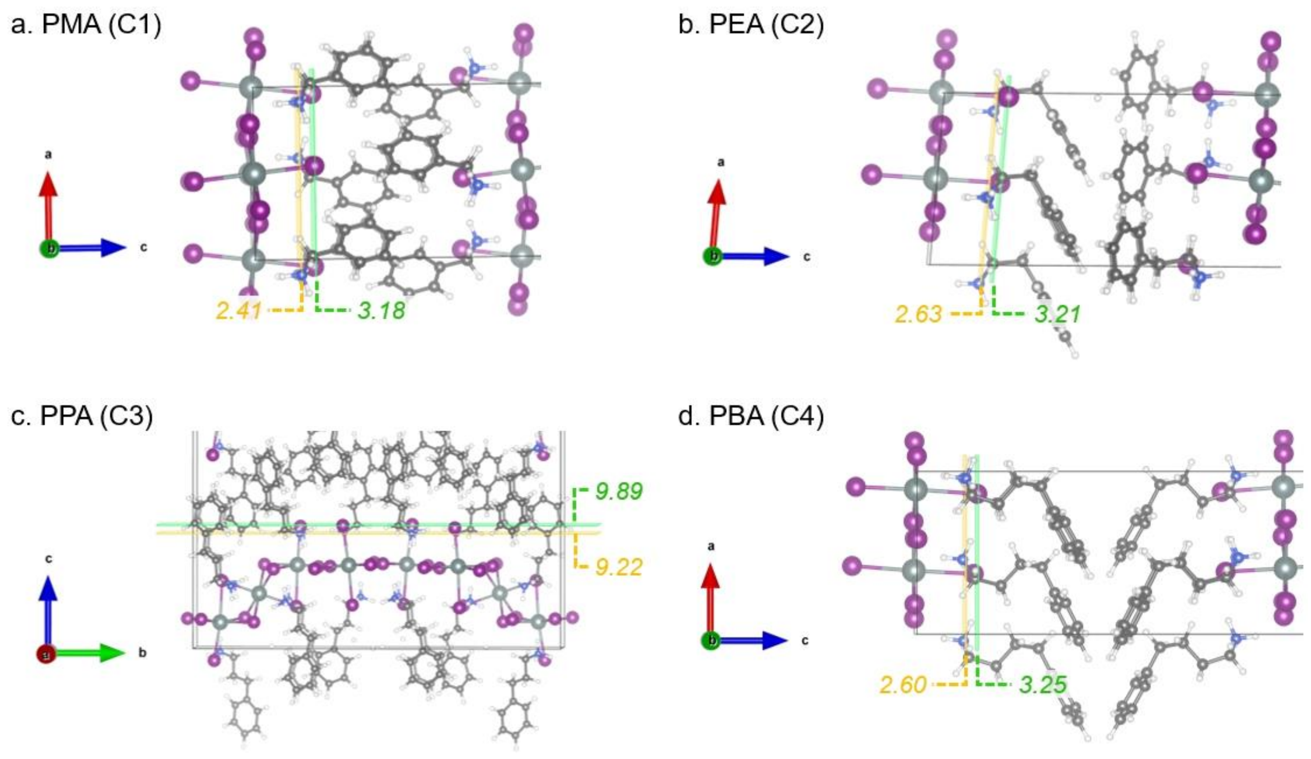

Figure S8. Organic cation penetration depth (d_P) across four perovskite systems discussed in the main manuscript. In each structure, the *001* lattice planes are illustrated at varying depths (Å) relative to the origin. The green plane contains the iodine (I) atoms in the inorganic layer, and the yellow plane indicates the position of nitrogen (N) atoms of the organic layer. The perpendicular distances between these planes, d_P, for the respective systems are: (a) 0.77 Å [PMA], (b) 0.58 Å [PEA], (c) 0.67 Å [PPA], and (d) 0.65 Å [PBA].

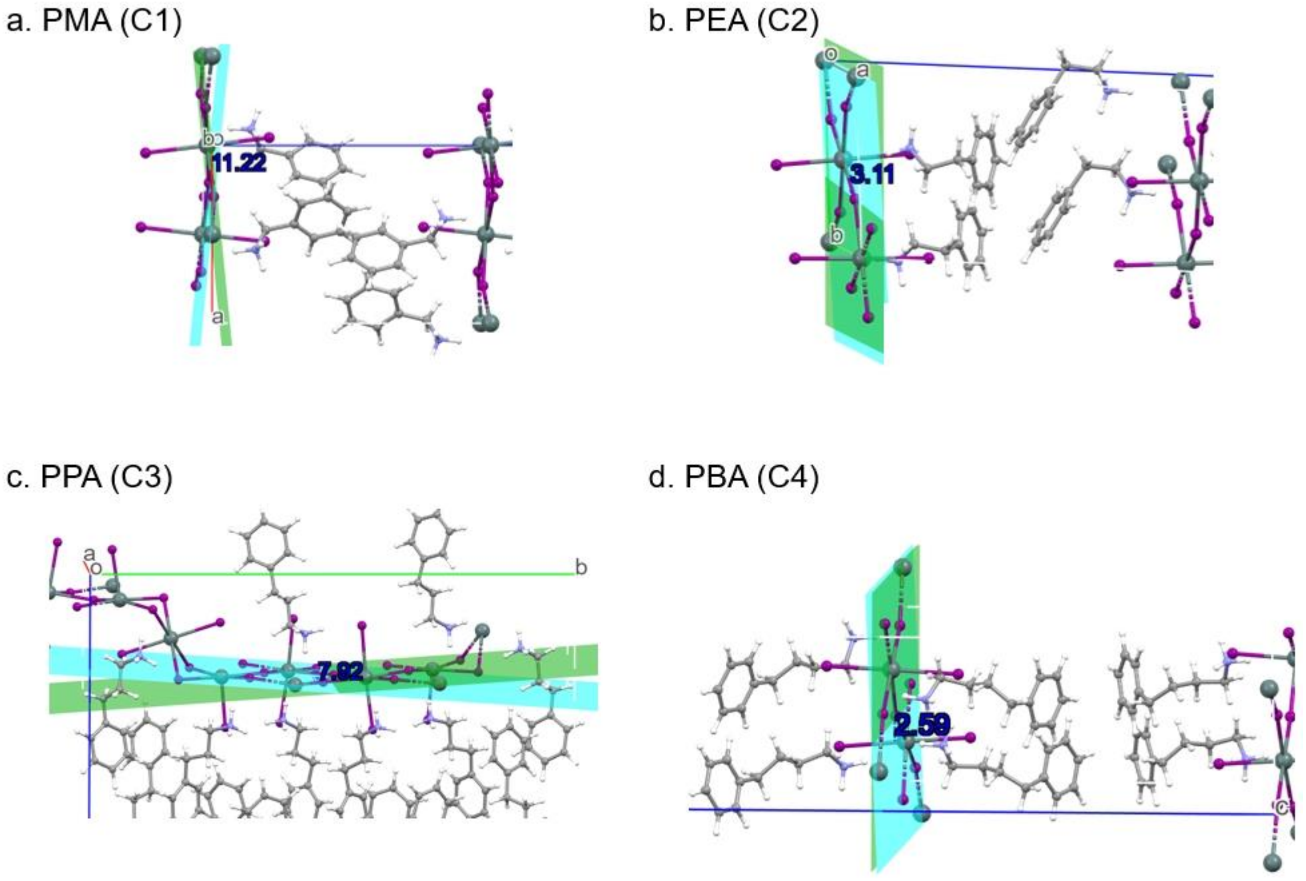

Figure S9. Inorganic layer distortion angle (θ, degrees) in four perovskite systems described in the main text. For each system, the planes containing the planar [SnI_4] units from two adjacent octahedra are depicted in green and cyan., The angle between these two planes, termed the distortion angle θ, quantifies the extent of structural distortion in each material. The measured distortion angles are: (a) 11.22° [PMA], (b) 3.11° [PEA], (c) 7.92° [PPA], and (d) 2.59° [PBA].

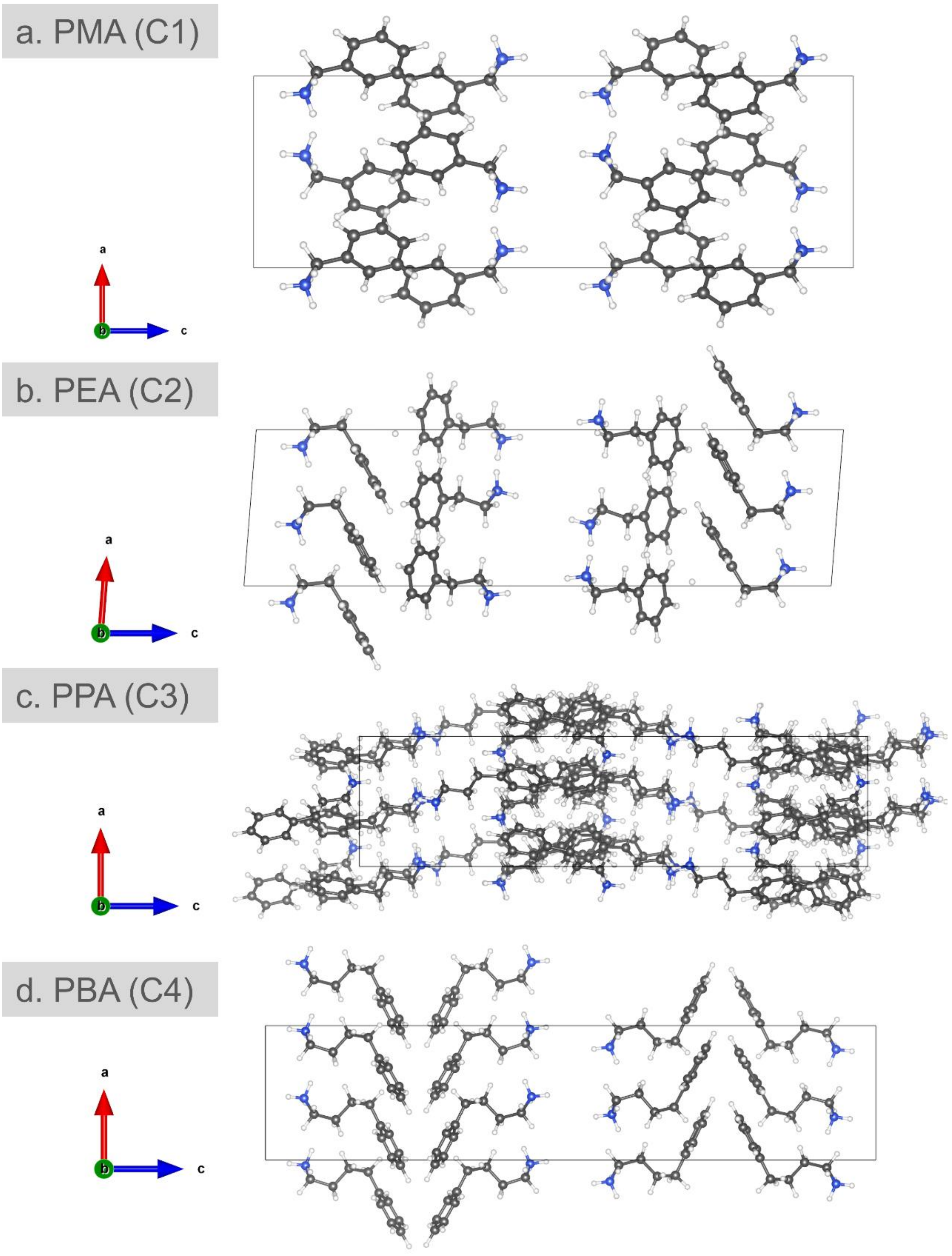

Figure S10. Orientation of organic spacers along the *ac* plane in the four investigated perovskite structures. The inorganic [SnI_6] layer lies within the *ab* plane and is excluded for clarity.

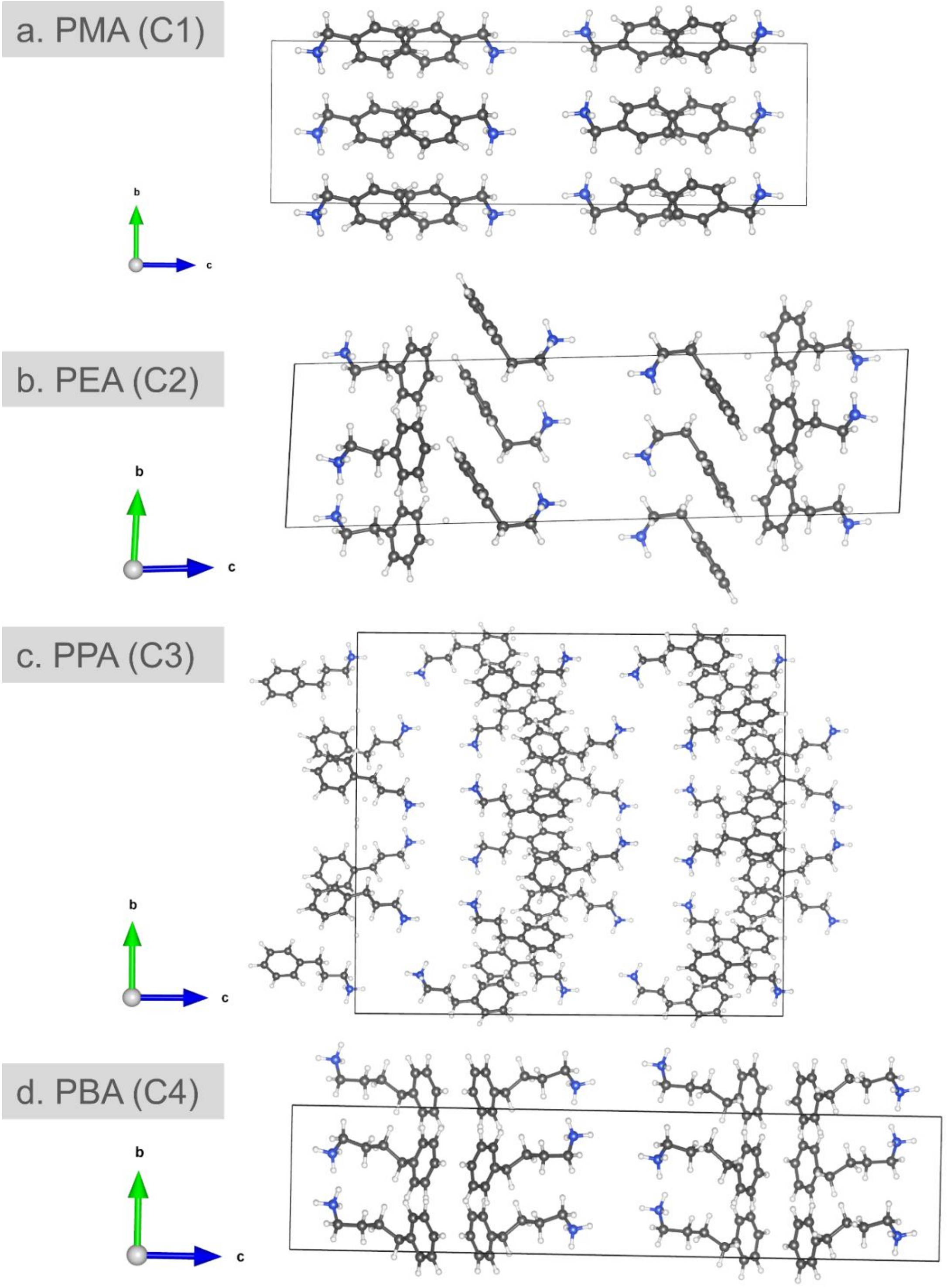

Figure S11. Orientation of organic spacers along the *bc* plane in the four investigated perovskite structures. The inorganic [SnI_6] layer lies within the *ab* plane and is excluded for clarity.

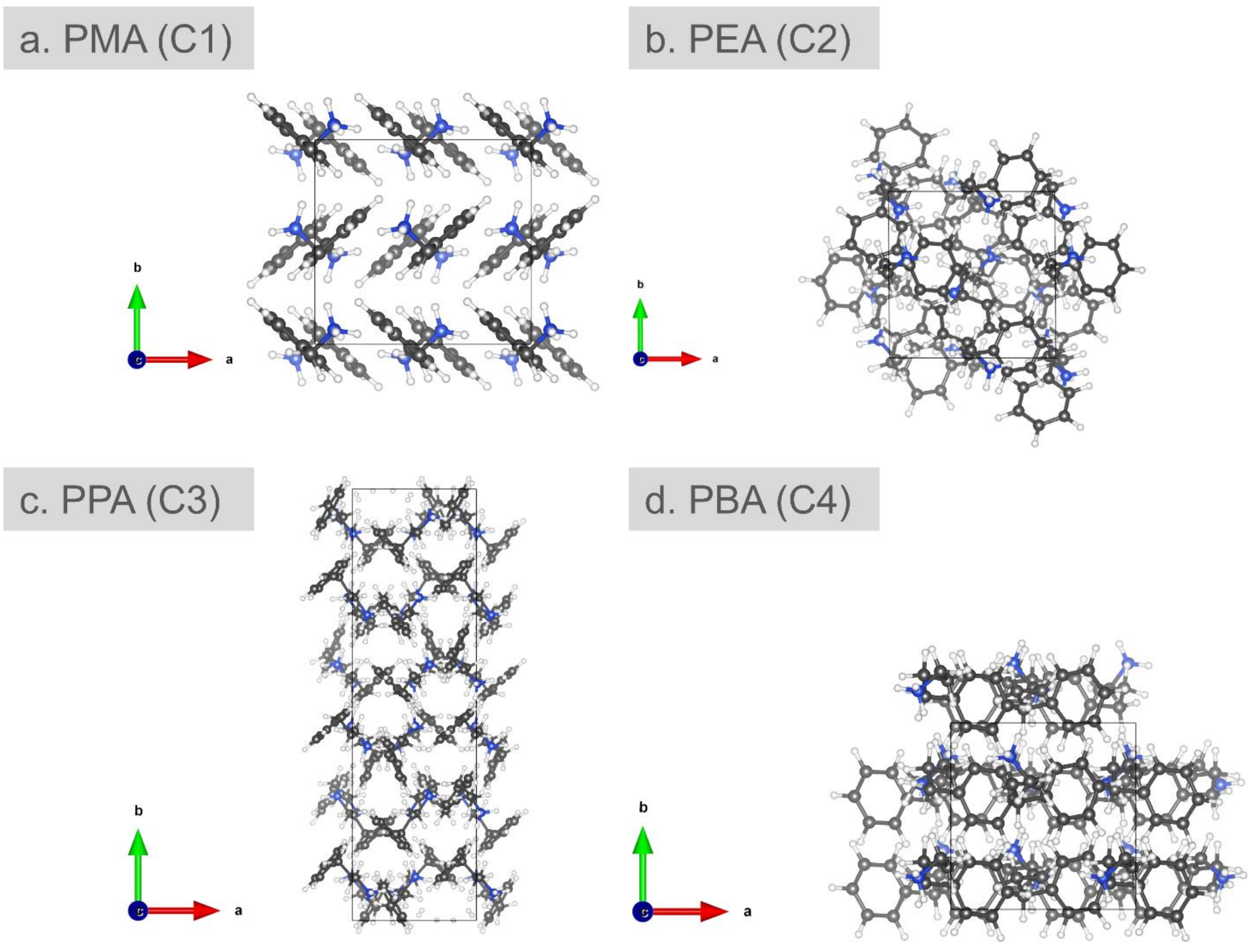

Figure S12. Orientation of organic spacers along the *ab* plane in the four investigated perovskite structures. The inorganic [SnI_6] layer lies within the *ab* plane and is excluded for clarity.

References

1. Choi, H. H., Cho, K., Frisbie, C. D., Sirringhaus, H., & Podzorov, V. (2018). Critical assessment of charge mobility extraction in FETs. Nature Materials, 17(1), 2-7.
2. Kresse, G.; Furthmüller, J. Efficient Iterative Schemes for Ab Initio Total-Energy Calculations Using a Plane-Wave Basis Set. Phys. Rev. B 1996, 54, 11169.
3. Perdew, J. P.; Burke, K.; Ernzerhof, M. Generalized Gradient Approximation Made Simple. Phys. Rev. Lett. 1996, 77, 3865.4
4. Grimme, S.; Antony, J.; Ehrlich, S.; Krieg, H. A Consistent and Accurate Ab Initio Parametrization of Density Functional Dispersion Correction (DFT-D) for the 94 Elements H-Pu. J. Chem. Phys. 2010, 132, 154104.
5. Kresse, G.; Furthmüller, J. Efficiency of Ab-Initio Total Energy Calculations for Metals and Semiconductors Using a Plane-Wave Basis Set. Comput. Mater. Sci. 1996, 6, 15;
6. Kresse, G.; Joubert, D. From Ultrasoft Pseudopotentials to the Projector Augmented-Wave Method. Phys. Rev. B 1999, 59, 1758.
7. Momma, K.; Izumi, F. VESTA 3 for Three-Dimensional Visualization of Crystal, Volumetric and Morphology Data. J. Appl. Crystallogr. 2011, 44, 1272.
8. Ganose M., Jackson A. J., A.; Scanlon O., Sumo D.: Command-Line Tools for Plotting and Analysis of Periodic Ab Initio Calculations. J. Open Source Softw. 2018, 3, 717.
9. Wang, V.; Xu, N.; Liu, J.-C.; Tang, G.; Geng, W.-T. VASPKIT: A User-Friendly Interface Facilitating High-Throughput Computing and Analysis Using VASP Code. Comput. Phys. Commun. 2021, 267, 108033.
10. Breiby, D. W.; Bunk, O.; Andreasen, J. W.; Lemke, H. T.; Nielsen, M. M. Simulating X-ray diffraction of textured films. J. Appl. Crystallogr. 2008, 41, 262–271.
11. Marcon, V., Breiby, D. W., Pisula, W., Dahl, J., Kirkpatrick, J., Patwardhan, S., ... & Andrienko, D. (2009). Understanding structure-mobility relations for perylene tetracarboxydiimide derivatives. Journal of the American Chemical Society, 131(32), 11426-11432.
12. Cho, E., Risko, C., Kim, D., Gysel, R., Cates, N., Breiby, D. W., ... & Bredas, J. L. (2012). Three-dimensional packing structure and electronic properties of biaxially oriented poly (2, 5-bis (3-alkylthiophene-2-yl) thieno [3, 2-b] thiophene) films. Journal of the American Chemical Society, 134(14), 6177-6190.

13. Mao, L., Tsai, H., Nie, W., Ma, L., Im, J., Stoumpos, C. C., ... & Kanatzidis, M. G. (2016). Role of organic counterion in lead-and tin-based two-dimensional semiconducting iodide perovskites and application in planar solar cells. Chemistry of Materials, 28(21), 7781-7792.
14. Ai, X., Beard, M. C., Knutsen, K. P., Shaheen, S. E., Rumbles, G., & Ellingson, R. J. (2006). Photoinduced charge carrier generation in a poly (3-hexylthiophene) and methanofullerene bulk heterojunction investigated by time-resolved terahertz spectroscopy. The Journal of Physical Chemistry B, 110(50), 25462-25471.
15. Cunningham, P. D., & Hayden, L. M. (2008). Carrier dynamics resulting from above and below gap excitation of P3HT and P3HT/PCBM investigated by optical-pump terahertz-probe spectroscopy. The Journal of Physical Chemistry C, 112(21), 7928-7935.
